# A subgroup of microsatellite stable colorectal cancers has elevated mutation rates and different responses to alkylating and oxidising agents

**DOI:** 10.1038/sj.bjc.6601740

**Published:** 2004-03-16

**Authors:** A R Parker, C P Leonard, L Hua, R O Francis, S Dhara, A Maitra, J R Eshleman

**Affiliations:** 1 Department of Pathology, Johns Hopkins University, Baltimore, MD 21205, USA; 2 Department of Oncology, Johns Hopkins University, Baltimore, MD 21205, USA

**Keywords:** microsatellite stable, *hprt*, mutator phenotypes, 6-thioguanine, colorectal cancer, MSI, MSS

## Abstract

An early step in the carcinogenesis of hereditary non-polyposis colorectal cancer (HNPCC) and some sporadic colorectal cancers (CRCs) is the acquisition of a ‘mutator phenotype’ resulting from defects in DNA mismatch repair (MMR) genes, which normally maintain genomic stability. This mutator phenotype causes an approximately 100–1000-fold increase in base substitutions and small insertion/deletion mutations thereby driving carcinogenesis. It also causes genome-wide microsatellite instability (MSI) due to the inability to repair mutations within these small, hard to replicate, repetitive DNA elements. In contrast, less is known about the role of mutator phenotypes in microsatellite stable (MSS) CRC. In this report, we have measured the mutation rates in 11 MSS CRC cell lines to obtain an estimate of the prevalence of mutator phenotypes in MSS carcinogenesis. Of the 11 cell lines, three of them (27%) possess spontaneous hypoxanthine phosphoribosyltransferase mutation rates approximately 10–100-fold above background. When challenged with alkylating and oxidising agents, the degree of survival and apoptotic responses are different, indicating that these cell lines may represent more than one mutator phenotype. These data demonstrate that a significant portion of MSS CRC cell lines has increased mutation rates and that this may play a role in MSS CRC carcinogenesis.

Since the low spontaneous mutation rate in normal cells is difficult to reconcile with the relatively large number of mutations observed in tumours (Fearon and Vogelstein, 1990), Loeb and Nowell have proposed that early in multistep carcinogenesis, cells first acquire a ‘mutator phenotype’ (Nowell, 1976; Loeb, 1991, 2001). Because of their increased mutation rates, cells with mutator phenotypes can produce requisite numbers of mutations in oncogenes and tumour suppressor genes, which are subsequently selected for and manifested in the final tumour (Nowell, 1976; Fearon and Vogelstein, 1990). Mutator phenotypes should therefore be thought of as carcinogenic because they accelerate mutation production during tumorigenesis. The same oncogenes and tumour suppressor genes that are mutated in the spontaneous pathway may be similarly involved in mutator phenotype pathways although the spectrum of mutations may be somewhat different (Lipton *et al*, 2003).

The hypothesis of the role for mutator phenotypes in tumorigenesis is supported by several human genetic disorders where mutations in genome caretaker genes have been shown to both elevate the cell's mutation rate and predispose to neoplasia. For example, xeroderma pigmentosum (XP)^4^ patients possess inherited defects in nucleotide excision repair (NER) and a predilection to skin cancer, and the mutation rate is significantly elevated in their cells when exposed to UV light (Maher *et al*, 1979). Cells from hereditary non-polyposis colorectal cancer (HNPCC) associated cancers and sporadic colorectal cancer (CRCs), possess a mutator phenotype that arises from genetic or epigenetic defects in the DNA mismatch repair (MMR) system (Fishel *et al*, 1993; Leach *et al*, 1993; Kane *et al*, 1997; Veigl *et al*, 1998). This mutator phenotype increases the spontaneous rates of base substitution and small insertion/deletion mutations approximately 100–1000-fold (Bhattacharyya *et al*, 1994, 1995; Eshleman *et al*, 1995, 1996) and provides a plausible explanation for the high rate of carcinogenesis observed.

We hypothesised that mutator phenotypes might occur commonly in microsatellite stable (MSS) CRC carcinogenesis and to test this, we screened a panel of MSS CRC cell lines. To test this hypothesis, we employed the classic selectable mutation marker for human studies, hypoxanthine phosphoribosyltransferase (HPRT). We report here that three out of 11 (3/11, 27%) MSS CRC cell lines tested possess elevated HPRT mutation rates. The MSS CRC cell lines with elevated mutation rates demonstrate unique patterns of sensitivity to the cytotoxic chemicals *N*-methyl-*N′*-nitro-*N*-nitrosoguanidine (MNNG) and hydrogen peroxide (H_2_O_2_). They are at least in part independent of the recently reported MutY mutations (Al-Tassan *et al*, 2002; Sieber *et al*, 2003). We propose that mutator phenotypes are common in MSS CRC, are likely more than one type, and may play an important role in MSS CRC carcinogenesis.

## MATERIALS AND METHODS

### Cell lines

The MSS CRC cell lines were either isolated and generously provided by Dr James KV Willson (Case Western Reserve University, those with Vaco prefixes) or purchased from the ATCC. Other than there being microsatellite stable, there were no other criteria for selecting these cell lines. One cell line was excluded because it had a low cloning efficiency.

### Cell culture, initial selection of 6-thioguanine (6TG) resistant mutants, and fluctuation analysis

Cells were grown and selected in 6TG (Sigma, St Louis, MO, USA) as published previously (Willson *et al*, 1987; Eshleman *et al*, 1995). Briefly, after subculturing, cells were counted and plated in 96-well plates to determine the cloning efficiency (CE) and at 10 000 cells per well in 1.5–5 *μ*g ml^−1^ 6TG to test for mutations. Plates were fed every 2 weeks and wells scored positive for growth by phase microscopy after 6 weeks. This was repeated until approximately 30 million clonogenic units had been analysed (three of the cell lines from the series, SW837, Vaco489 and Vaco576, have been preliminarily analysed, but with only approximately 5 million clonogenic units (Eshleman *et al*, 1995)).

Cell lines exhibiting mutations in the initial screen were examined using fluctuation analysis as previously described (Luria, 1943; Eshleman *et al*, 1995). Briefly, cells were purged of pre-existing mutants by dilution and regrowth from 100 cells in 10 replicate cultures. These cultures were independently expanded to approximately 10–30 million cells and plated for cloning efficiency and mutation frequency as described.

### Determination of cloning efficiency and calculation of mutation rates and frequencies

CE was determined by seeding three and 10 cells per well in 96-well plates in the presence of SW480 or native feeder cells lethally irradiated with 8000 centi-Gy ^137^Cs at a concentration of 10 000 cells per well. CE was calculated using Poisson statistics as described previously (Furth *et al*, 1981), where CE=[−ln (fraction of negative wells)]/(number of cells seeded per well). The CE for each cell line presented in Table 1

**Table 1:** Mutation frequencies and mutation rates of MSS CRC lines

**Name**	**CE (%)**	**No. of cells plated**	**No. of CFUs selected**	**MF**	**MR**
*Mutators*					
SW948	23.0	97.4 M	22.4 M	20.4±7.6	40.0
Vaco411	32.2	47.9 M	15.4 M	77.0±13.8	157.0
Vaco8	69.0	28.8 M	19.9 M	38.6±15.0	90.0
*Nonmutators*					
CACO2	19.5	176.1 M	34.2 M	<0.3	<0.1
HT29	52.2	66.5 M	34.7 M	<0.3	<0.1
COLO205	32.8	111.3 M	36.5 M	<0.3	<0.1
SW837	14.0	237.9 M	33.3 M	<0.3	<0.1
SKCO1	20.5	149.8 M	30.7 M	<0.3	<0.2
Vaco364	52.2	58.4 M	30.5 M	<0.3	<0.1
Vaco489	10.8	276.4 M	29.8 M	<0.3	<0.1
Vaco576	34.2	96.5 M	33.0 M	<0.3	<0.1

CE=cloning efficiency; CFU=colony-forming units; MF=mutation frequency; MR=mutation rate. The CE for each cell line presented here is expressed as the mean for all replicate cultures of that line. No. of CFUs selected is the total number of CFUs plated into 6TG for assay of HPRT mutants. M=10^6^. Mutation frequencies are expressed as 10^−7^ mutants per CFU. Mutation rates are expressed as 10^−8^ mutations per locus per generation (Eshleman *et al*, 1995).

is expressed as the mean for all replicate cultures of that line. Mutation frequency (MF) and mutation rate (MR) calculations were performed essentially as described (Eshleman *et al*, 1995). Briefly, mutation rates were calculated using Poisson corrected counts (Furth *et al*, 1981) and the tables of Capizzi and Jameson (Capizzi and Jameson, 1973). For cell lines lacking mutations, mutation frequencies and rates were calculated assuming that one hypothetical mutant had been isolated and expressed as less than that value.

### Microsatellite analysis

To test for microsatellite instability, cells were plated at 1–3 cells per well, in 96-well plates, and expanded. In total, 10 independent clones, per cell line, were analysed for microsatellite length using the five nucleotide repeats recommended at the NCI-sponsored conference on microsatellite instability (MSI) for CRC (Boland *et al*, 1998) BAT25, BAT26, D5S346, D2S123 and D17S250 using multiplex PCR and capillary electrophoresis (Berg *et al*, 2000).

### *hprt* cDNA sequencing

To confirm the presence of mutations, RNA from 6TG resistant colonies was isolated using the RNAgents Total RNA Isolation System (Promega, Madison, WI, USA). The isolated RNA (approximately 500 ng) was reverse-transcribed using 5 U AMV reverse transcriptase (Boehringer Mannheim, Mannheim, Germany) in a 20 *μ*l buffered solution with 20 U of RNasin (Promega), 0.5 *μ*g of Oligo (dT)_12–18_ primer (GIBCO, Grand Island, NY, USA), and 20 mM of dNTPs. After the incubation, 5 *μ*M of M13 tailed forward sense primer S-27 (−27 to −12 relative to the AUG) 5′-GTAAAACGACGGCCAG-TCAGCCCGCGCGCCGGC-3′, 5 *μ*M of M13 tailed reverse primer AS661 (661-557) 5′-CAGGAAACAGCTATGAC-TCAACTTGAACTCTC-3′ and 1 *μ*l of template DNA were added to the Taq PCR Master Mix (Qiagen, Valencia, CA, USA) and incubated for 10 min at 94°C. The cDNA was amplified for a total of 30 cycles with each cycle consisting of 1 min at 94°C, 1 min at 60°C and 3 min at 72°C. Final extension of the PCR amplified product was for 10 min at 72°C. PCR amplified samples were sequenced using M13 primers and Big Dye cycle sequencing on an ABI3700 sequencer (Applied Biosystems, Foster City, CA, USA).

### Cell viability after treatment with *N*-methyl-*N*′-nitro-*N*-nitrosoguanidine (MNNG) and hydrogen peroxide (H_2_O_2_)

For the cell viability assay, exponentially growing cells were trypsinised and washed twice in serum-free minimal essential medium (SF-MEM). Washed cells were suspended in SF-MEM and treated with 0–5 *μ*M MNNG (Sigma) for 45 min at 37°C or with 0–300 *μ*M H_2_O_2_ (Sigma) for 60 min at 37°C. After treatment, the cells were washed once with MEM2+media (Willson *et al*, 1987), resuspended in fresh growth medium, and seeded (approximately 500 000 cells well^−1^ (200 000 for Vaco8) in six-well plates. The cells were grown for 5 days (for H_2_O_2_) or 6 days (for MNNG), subcultured and counted using trypan blue exclusion with a haemocytometer. Experiments were performed three times, each in triplicate.

### Assessment of apoptotic response

Apoptosis was assessed after treating cells with either_5 *μ*M MNNG or 300 *μ*M H_2_O_2_ as described above, and monitoring the cells at various time points after treatment. Apoptosis was determined as described (Wang *et al*, 1995) using DAPI as the vital dye and propidium iodide as the excluded dye. Briefly, cells were washed at various times after treatment and stained in MEM with 112 *μ*g ml^−1^ DAPI (Sigma) for 10 min, followed by 60 *μ*g ml^−1^ propidium iodide (Sigma) and fluorescence microscopy. In all, 200 cells were counted and the percent of cells that demonstrated apoptotic bodies calculated.

## RESULTS

### Three out of 11 MSS CRC cell lines possess elevated HPRT mutation rates

**Figure 1:**
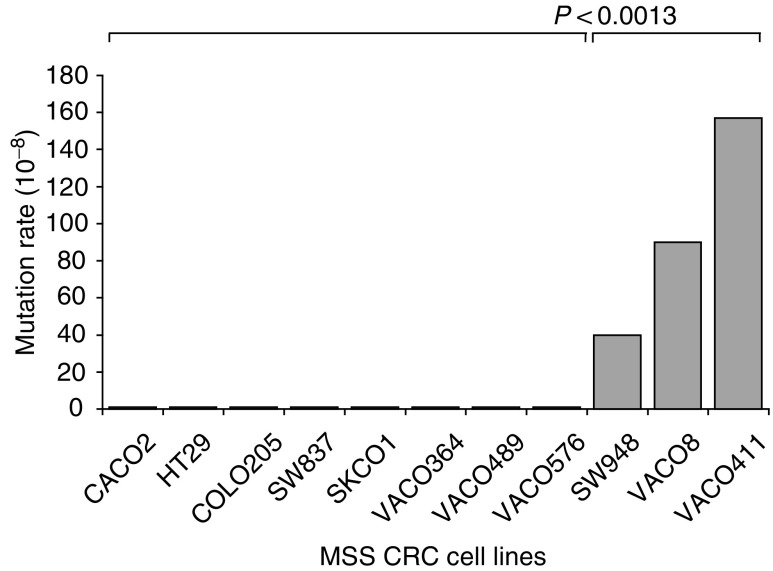
Mutation rates of the MSS CRC cell lines Vaco8, Vaco411 and SW948 are increased. Mutation rates in the 11 MSS CRC cell lines as described. The mutators were compared to the nonmutators using the unpaired Student's *t*-test.

We measured the spontaneous HPRT mutation rate in 11 MSS CRC cell lines (Table 1, and Figure 1

). For initial screening, these cell lines were expanded and plated in the presence of 6TG, until approximately 30 million colony-forming units (CFUs) had been tested for 6TG resistance. Since many of the cell lines displayed no mutants, we estimated the rate using a single hypothetical mutant. The mutation rates of eight of the MSS CRC cell lines were <0.1–0.2 × 10^−8^ mutations per locus per cell division (Table 1) and defined the baseline level of spontaneous mutation. We previously reported a limited analysis of three of the nonmutator cell lines (Eshleman *et al*, 1995), that we have analysed to a much greater degree in the present study. To obtain the best estimate of the overall prevalence of elevated mutation rates in MSS CRC, we included the previously identified MSS CRC cell line Vaco411, since it was part of an original series of sequential cell lines expanded and examined in more detail in this report (Eshleman *et al*, 1998). During the initial screen in 6TG, while most of the cell lines yielded no HPRT mutants (Table 1, Figure 1), three cell lines possessed an elevated mutation rate (approximately 100-fold above background nonmutator cell lines). Statistical analysis confirmed that the elevated mutation rates in these three MSS CRC cell lines are significantly different from the baseline mutation rates in the nonmutator CRC cell lines (*P*=0.0013, unpaired *t*-test). Furthermore, the mutation frequency of one of them (Vaco411) appears to be higher than the other two. Limited sequencing of the *hprt* cDNA (Eshleman *et al*, 1996) from several mutants from each of the cell lines exhibiting 6TG resistance confirmed that mutations within *hprt* were indeed present (manuscript in preparation). The ages of the patients when the tumours were diagnosed were 32, 56 and 81 years old for Vaco411, Vaco8 and SW948, respectively.

### The MSS CRC cells with elevated mutation rates are differentially sensitive to alkylation and oxidative chemical challenge

**Figure 2:**
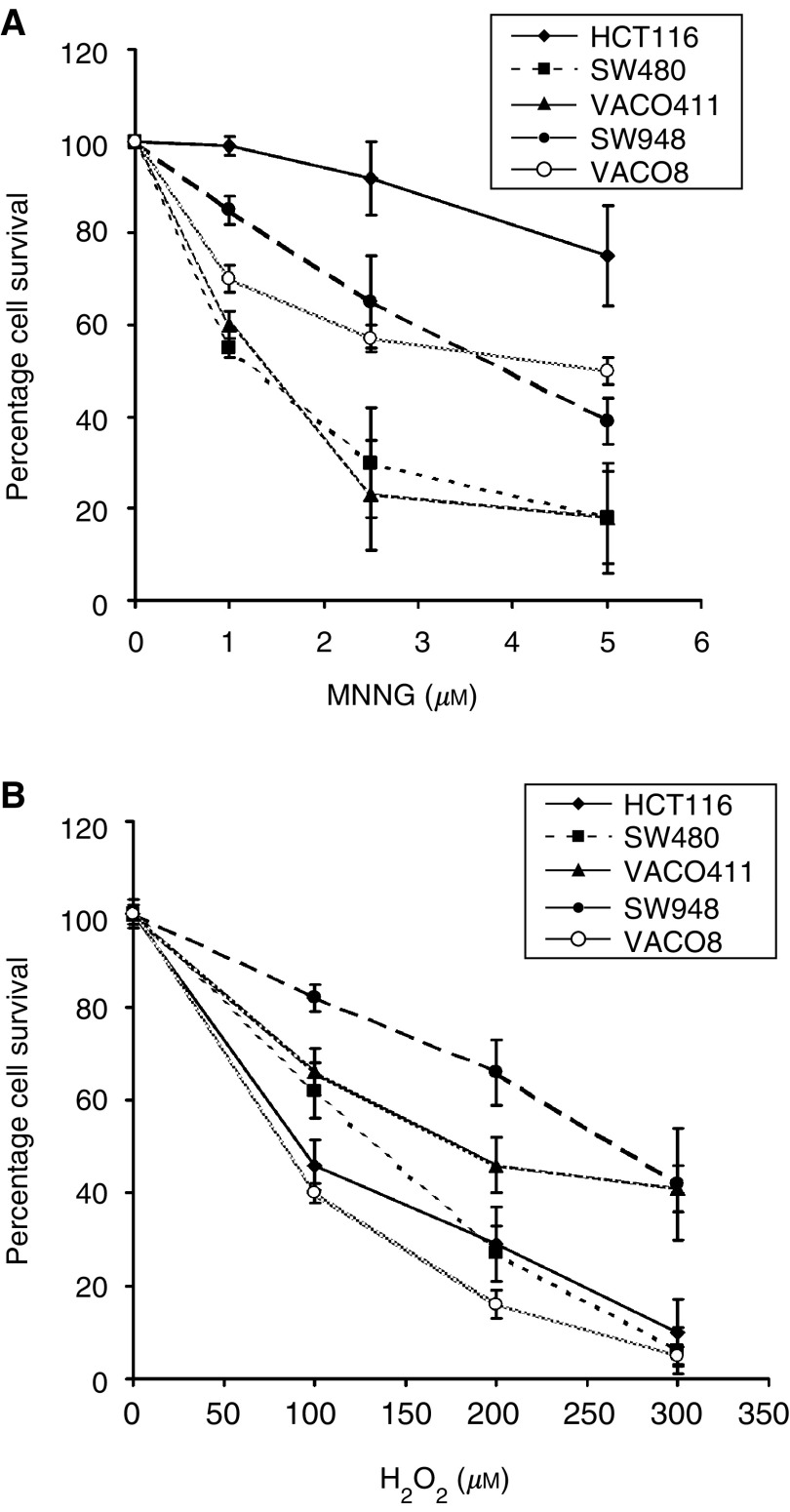
Cell survival following alkylating and oxidative chemical challenge. (**A**) Vaco8 and SW948, but not Vaco411, possess intermediate resistance to MNNG. The three MSS mutators and two control cell lines (HCT116, MSI mutator and SW480, MSS nonmutator) were treated with 0–5 *μ*M MNNG for 45 min at 37°C. The cells were grown for 6 days, subcultured and counted using trypan blue exclusion with a haemocytometer. (**B**) Vaco411 and SW948, but not Vaco8, possess resistance to H_2_O_2_. The same cell lines were treated with 0–300 *μ*M H_2_O_2_ for 60 min at 37°C. The cells were grown for 5 days, subcultured and counted using trypan blue exclusion with a haemocytometer. Experiments were performed three times, each in triplicate and the error bars represent 1 s.e.m.

A consistent feature of cells with elevated mutation rates due to defective MMR has been the tolerance to alkylating agents such as MNNG (Branch *et al*, 1993; Kat *et al*, 1993). We therefore asked whether the MSS CRC cell lines with mutator phenotypes would be altered in their responses to MNNG. As demonstrated previously (Koi *et al*, 1994), the MMR deficient cell line, HCT116, was relatively resistant to the cytotoxic effects of MNNG since this cell line carries two defective *mlh1* genes (Figure 2A

, (Papadopoulos *et al*, 1994)). In contrast and as expected, the SW480 MMR proficient cell line was sensitive to the cytotoxic effects of MNNG since it possesses a functional MMR system. Of the MMR proficient mutators, Vaco411, was sensitive to MNNG similar to SW480 (approximately 18% cell viability after treatment with 5 *μ*M MNNG), while SW948 and Vaco8 were both intermediate, demonstrating more resistance than both SW480 and Vaco411 but less than HCT116 (compare 18% of cells remaining after 5 *μ*M MNNG, to 39% for SW948, 50% for Vaco8 and 75% for HCT116).

We have previously demonstrated that two of the three mutators have elevated levels of the mutagenic base 8-oxoG (Parker *et al*, 2002). Since both Vaco411 and Vaco8 possesses low rates of 8-oxoG repair and elevated genomic 8-oxoG levels (Parker *et al*, 2002), we hypothesised that a mutator phenotype, which affects 8-oxoG repair, might affect their ability to repair oxidative DNA damage induced by hydrogen peroxide (H_2_O_2_). Figure 2B shows that both Vaco411 and the MutY proficient cell line SW948 were more resistant towards H_2_O_2_ treatment (41 and 42% of cells remaining respectively after 300 *μ*M H_2_O_2_) than the other three cell lines.

### Two of the three mutator cell lines undergo apoptotic cell death after chemical challenge

**Figure 3:**
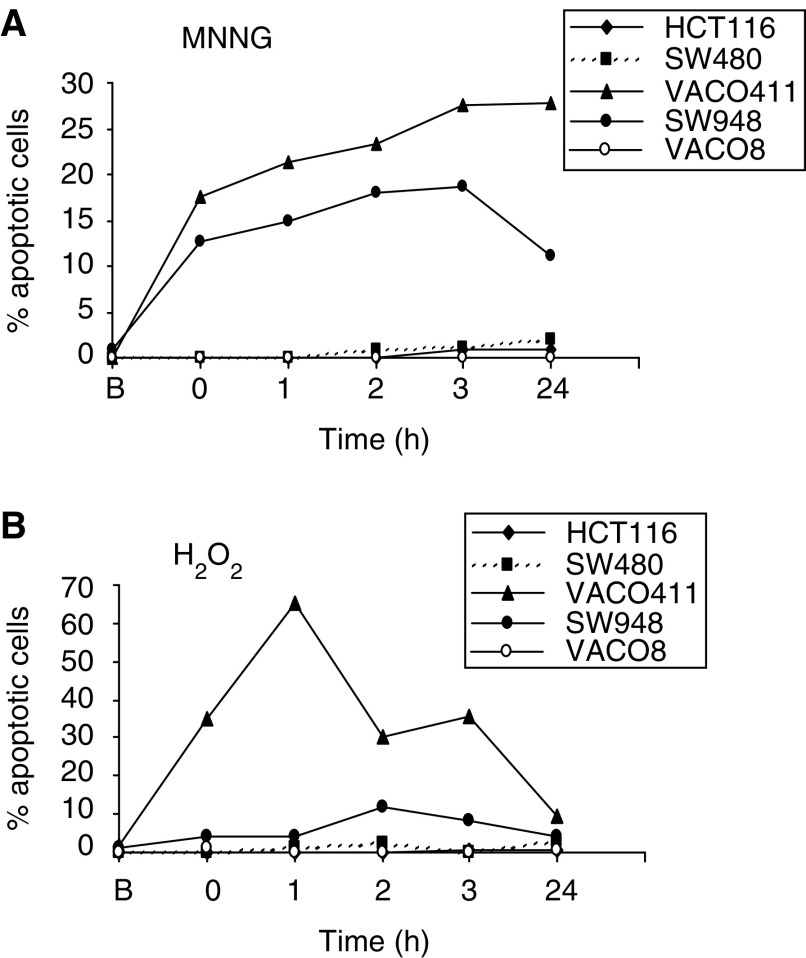
Two of the three MSS mutator cell lines demonstrate apoptotic response to chemical challenge. Apoptotic response after treatment with MNNG (**A**) or H_2_O_2_ (**B**). Cells either before (**B**) or after treatment with the maximal dose of drug, were recovered for various times and the percent of cells undergoing apoptosis determined by nuclear staining (see Materials and methods).

Having demonstrated this differential toxicity, we next asked whether the cell death occurred through apoptosis. We challenged the cell lines with either MNNG or H_2_O_2_ at the highest doses tested above, and monitored the cells for apoptosis after 0, 1, 2, 3 and 24 h after treatment. Harvested cells were stained with DAPI and propidium iodide and directly scored for the presence of apoptotic bodies by fluorescence microscopy (see Materials and Methods (Wang *et al*, 1995)). When challenged with MNNG, two of the mutator cell lines (Vaco411 and SW948) showed significant apoptosis, while the other mutator (Vaco8) and the MMR proficient and deficient controls demonstrated little, if any, apoptosis (Figure 3A

). In response to peroxide treatment, the same two mutator lines exhibited apoptosis, although the degree of apoptosis and the timing of it varied from that seen in response to MNNG (Figure 3B). Again, the other three cell lines demonstrated little, if any, apoptosis.

### The three MSS CRC cell lines with elevated mutation rates are microsatellite stable

To confirm that the elevated mutation rates were not due to defective MMR activity and that the cell lines possess stable microsatellite DNA, a panel of five microsatellite markers, two mononucleotides and three dinucleotides, were analysed. In all, 10 independently derived clones, from each of the three MSS mutator cell lines and HCT116 as a positive control, were analysed using these five microsatellite markers recommended at a US NCI-sponsored conference on MSI for CRC (Boland *et al*, 1998) BAT25, BAT26, D5S346, D2S123 and D17S250 using multiplex PCR and capillary electrophoresis (Berg *et al*, 2000). All five loci that were PCR amplified and had stable lengths confirming that Vaco411, Vaco8 and SW948 are microsatellite stable (data not shown) and therefore likely MMR competent. For Vaco411, we have previously reported that its mutation spectrum is inconsistent with an MMR defect and that it is functionally MMR competent when directly challenged with mispaired DNA substrates (Eshleman *et al*, 1998b).

## DISCUSSION

The current study suggests that mutator phenotypes may play a significant overall role in MSS CRC carcinogenesis since, from a panel of 11 MSS CRC cell lines, three possessed elevated mutation rates (27%). The majority (eight out of 11, 73%) however, showed normal baseline rates.

In MMR-deficient CRC, the spontaneous mutation rates are generally elevated at least 100–1000-fold above the baseline normal rate. In contrast, the increased HPRT mutation rates we report in this current study in the MSS CRC cell lines are approximately 10–100-fold increased relative to control levels in nonmutator MSS CRC cells and accordingly are designated as ‘intermediate’ elevated levels. It is noteworthy that intermediate mutation rates are present in other diseases such as Bloom's syndrome (approximately 10-fold higher than normal patients) (Warren *et al*, 1981) and Werner syndrome (10–50-fold higher than normal cell lines) (Fukuchi *et al*, 1989), and we interpret the intermediate elevated mutation rates observed here as likely biologically significant.

Given the differences in mutation rates, the differential responses to MNNG and H_2_O_2_, and the differences in the levels of genomic 8-oxoG, it seems likely that there is more than one underlying defect responsible for these novel MMR-independent mutator phenotypes. The cell line characteristics from this study are shown in Table 2

**Table 2:** Characteristics of MSS CRC mutator cell lines

			**Cytotoxic response**	
**Cell line**	**Microsatellite status**	**Mutator defect**	**H_2_O_2_**	**MNNG^a^**
Vaco8	MSS	Elevated 8-oxoG	Normal	Int. resistant
Vaco411	MSS	Elevated 8-oxoG	Resistant	Normal
SW948	MSS	Unknown	Resistant	Int. resistant
				
HCT116	MSI	mlh1	Normal	Resistant
SW480	MSS	None	Normal	Normal

a Int. indicates intermediate.

. Vaco8 possesses elevated levels of genomic 8-oxoG and low levels of MutY protein which may explain the elevated mutation rate since yeast and *Escherichia coli* (*E.coli*) defective in *mutY* possess elevated mutation rates approximately 30-fold above the background rate (Nghiem *et al*, 1988; Chang and Lu, 2002). However, Vaco8 possessed intermediate resistance to MNNG, but was appropriately sensitive to H_2_O_2_, suggesting instead that repair of alkylated DNA damage may be impaired. A partially impaired MMR system, one which can maintain normal microsatellite length but cannot detect or signal for apoptosis could be responsible, but it is also possible that intermediate level of resistance to MNNG and oxidative damage may be due to defects in another DNA repair system. Recently levels of *smad4/dpc4*, a transcription factor frequently lost in pancreatic cancer, have been shown to be defective in this cell line (Fink *et al*, 2003).

Vaco411, similar to Vaco8, also possesses elevated levels of genomic 8-oxoG and low levels of MutY protein, however this cell line is relatively resistant to H_2_O_2_ and unlike SW948, HCT116 and Vaco8, it was fully sensitive to MNNG suggesting that repair of oxidative DNA damage may be the more important defect. However, the spectrum of mutations in this cell line (Eshleman *et al*, 1998) is not fully explained by a single defect in MutY (Al-Tassan *et al*, 2002; Jones *et al*, 2002; Sieber *et al*, 2003), strongly suggesting that a second gene defect may also be present.

SW948, unlike the other four cell lines, is relatively resistant to both H_2_O_2_ and MNNG. The cell viability studies with MNNG suggest that although its MMR system is sufficient to maintain stable microsatellite length, it may not provide fully appropriate sensitivity to the methylating agent MNNG, as previously reported (Claij and Te Riele, 2002) and similar to that suggested for Vaco8 (Table 2). Resistance to oxidative stress, is also potentially consistent with an altered but semifunctional MMR system since *msh2*−/− (mut S homolog 2) mouse cells contain increased levels of oxidative DNA damage (DeWeese *et al*, 1998) and MMR-deficient cells can be somewhat resistant to H_2_O_2_ (Glaab *et al*, 2001). The spectrum of mutations in these novel MSS mutator phenotypes will likely provide insight to identify the genes responsible and comprehensive sequencing of the *hprt* cDNA from the mutator cell lines is currently underway.

It is interesting that the majority of cell lines (eight out of 11, 73%) did not possess elevated *hprt* mutation rates. There are three potential possibilities for this: (1) There is a mutator phenotype but it was not detected. This seems unlikely since the HPRT assay detects such a wide range of mutations (Albertini, 2001) (though not changes in chromosome number, see possibility #3 below). Further, we have established that when X-ray-induced HPRT mutant cells are spiked into wild-type cells, they are efficiently recovered (Eshleman *et al*, 1995). (2) There was a functional mutator phenotype expressed in these cells early during carcinogenesis, but it was transient and by the time of diagnosis and resection, the pre-existing mutator phenotype is no longer present, for example, due to transient methylation (Loeb, 2001). (3) Finally, there is in fact no functional mutator phenotype in the majority of MSS CRCs during carcinogenesis. This is consistent with the results of () although the cell lines and assays employed are substantially different.

One problem with possibility #3 is that most investigators consider genomic instability the fundamental (or at least one fundamental) feature of malignancy, because it explains how malignant cells acquire many of the critical features that distinguish them from their normal counterparts (e.g. ability to metastasise and acquire resistance to chemotherapeutics). One possible explanation is that most of the cell lines without a functional HPRT mutator phenotype manifest the chromosome instability (CIN) phenotype (Lengauer *et al*, 1997; Eshleman *et al*, 1998b). Since the CIN phenotype is assayed by centromeric fluorescence *in situ* hybridisation, it reports for changes in chromosome number whereas the HPRT assay is not expected to detect such changes. However, because CIN (production of aneuploidy) occurs at substantial rates, it can easily produce the second hit in tumour suppressor genes through loss of heterozygosity, and is therefore probably appropriate to consider CIN a mutator phenotype (Cahill *et al*, 1998).

In conclusion, these data demonstrate that a significant subgroup (approximately one-quarter) of MSS CRCs exist with elevated mutation rates that are likely independent of one another. This suggests that mutator phenotypes may also play a role in MSS CRC carcinogenesis.
